# Metabolic Influences of Gut Microbiota Dysbiosis on Inflammatory Bowel Disease

**DOI:** 10.3389/fphys.2021.715506

**Published:** 2021-09-27

**Authors:** Salma Sultan, Mohammed El-Mowafy, Abdelaziz Elgaml, Tamer A. E. Ahmed, Hebatoallah Hassan, Walid Mottawea

**Affiliations:** ^1^Faculty of Health Sciences, School of Nutrition Sciences, University of Ottawa, Ottawa, ON, Canada; ^2^Department of Microbiology and Immunology, Faculty of Pharmacy, Mansoura University, Mansoura, Egypt; ^3^Department of Microbiology and Immunology, Faculty of Pharmacy, Horus University, New Damietta, Egypt; ^4^Department of Cellular and Molecular Medicine, Faculty of Medicine, University of Ottawa, Ottawa, ON, Canada; ^5^Department of Biotechnology, Institute of Graduate Studies and Research, Alexandria University, Alexandria, Egypt

**Keywords:** IBD, gut microbiota, dysbiosis, SCFAs (short chain fatty acids), metabolome

## Abstract

Inflammatory bowel diseases (IBD) are chronic medical disorders characterized by recurrent gastrointestinal inflammation. While the etiology of IBD is still unknown, the pathogenesis of the disease results from perturbations in both gut microbiota and the host immune system. Gut microbiota dysbiosis in IBD is characterized by depleted diversity, reduced abundance of short chain fatty acids (SCFAs) producers and enriched proinflammatory microbes such as adherent/invasive *E. coli* and H_2_S producers. This dysbiosis may contribute to the inflammation through affecting either the immune system or a metabolic pathway. The immune responses to gut microbiota in IBD are extensively discussed. In this review, we highlight the main metabolic pathways that regulate the host-microbiota interaction. We also discuss the reported findings indicating that the microbial dysbiosis during IBD has a potential metabolic impact on colonocytes and this may underlie the disease progression. Moreover, we present the host metabolic defectiveness that adds to the impact of symbiont dysbiosis on the disease progression. This will raise the possibility that gut microbiota dysbiosis associated with IBD results in functional perturbations of host-microbiota interactions, and consequently modulates the disease development. Finally, we shed light on the possible therapeutic approaches of IBD through targeting gut microbiome.

## Introduction

Inflammatory Bowel Disease (IBD) is a chronic and remitting disorder characterized by relapsing episodes of gastrointestinal inflammation (Ramos and Papadakis, 2019). IBD is comprised of two major classes; ulcerative colitis (UC) and Crohn’s disease (CD). The inflammation in UC is restricted to the mucosal layer, leading to superficial damage of the bowel wall, while CD is associated with inflammation of all layers of the bowel wall (Kobayashi et al., 2020). Epidemiological studies showed that IBD is spreading widely throughout the world leading to a public health challenge worldwide (Zheng H. et al., 2017; Windsor and Kaplan, 2019; Kaplan and Windsor, 2021). It is widely accepted that the IBD pathogenesis results from an interplay between gut microbiota and the host immune system with the predisposition of genetic susceptibility and environmental factors (Ma et al., 2019; O’Donnell et al., 2019; Ramos and Papadakis, 2019; Pang et al., 2021). The microbiome-immune system interaction leads to improper immune activation responsible for the clinical and endoscopic observations in IBD patients (Ramos and Papadakis, 2019; Zheng et al., 2020).

The human gastrointestinal tract comprises very rich and diverse microbial community that includes more than 100 trillion microorganisms (Thursby and Juge, 2017). The gut microbiome plays an important role in human health and in the development of several chronic diseases. The altered composition of the gut microbiota (dysbiosis), in addition to the disturbance of the metabolic harmony of such microbial community plays a crucial role in the pathogenesis of IBD (Nishida et al., 2018; Zuo and Ng, 2018; Khan et al., 2019). Indeed, recent advances in molecular biology techniques together with improving the usability of microbial databases led to comprehensive characterization of microbial communities and revealing the association between gut microbiota dysbiosis and IBD (Wright et al., 2015; Lloyd-Price et al., 2019; Loeffler et al., 2020; Ryan et al., 2020). However, the actual contributions of this dysbiosis to the inflammation and the cause/effect relationship between gut microbe and IBD are still unclear.

In this review, we outline the recent findings that correlate gut microbiota dysbiosis and metabolic dysfunctionality to IBD. We highlight the metabolic pathways by which microbial dysbiosis could contribute to the inflammation seen in IBD. We also consider the connection among different metabolic pathways in relation to disease progression. In addition, we clarify the strategies of manipulating gut microbiota to promote gut health in IBD.

## Normal Gut Microbiota

Our gut is populated by a complex and dynamic microbial ensemble, which is considered an additional organ of the human body and collectively known as the gut microbiota. This cohort consists mainly of bacteria with low percentage of archeae, eukaryotic and viral members (Qin et al., 2010). Using fecal samples from 124 Europeans and Illumina-based metagenomic sequencing, the gut microbial gene catalog was estimated to be 100–150-fold that of human genes with 99.1% of bacterial origin. The bacterial species that compose the entire community were calculated to range from 1,000 to 1,150 prevalent species with at least 160 species per individual (Qin et al., 2010).

Studying mucosal and luminal microbiota structure via sequencing *16S rDNA* clones has revealed that approximately 90% of gut microbes are related to main two phyla; Firmicutes (51%) and Bacteroidetes (48%), with the remaining 10% distributed among Proteobacteria, Actinobacteria, Fusobacteria, and Verrucomicrobia (Eckburg et al., 2005). At lower levels, the majority of the detected Firmicutes were related to the Clostridia class, with the vast majority classified under clostridial clusters IV, XIVa, and XVI. Bacteroidetes, on the other hand, has showed less diversity than Firmicutes with 67% of its sequences classified under 3 main phylotypes*B. vulgatus*, *Prevotellaceae*, and *B*. *thetaiotamicron*. *B. thetaiotamicron* was the only common Bacteroidetes among all tested individuals. Other low abundant species related to other phyla included Proteobacteria species (*Sutterellawadsworthensis*, *Desulfomonaspigra*, and *Bilophilawadsworthia*), Actinobacteria genera (e.g., *Actinomyces*, *Collinsella*, and *Bifidobacterium*) and the mucin-degrading Verrucomicrobia species, *Akkermansiamuciniphila* (Eckburg et al., 2005). Along with bacteria, the most common coexisting fungal genera in the gut are *Saccharomyces*, *Candida*, *Galactomyces*, *Pleospora*, *Bullera*, *Aspergillus*, *Trametes*, *Sclerotinia*, *Penicillium*, and *Rhodotorula*, while virome mainly exists in the form of bacteriophages (Ungaro et al., 2019a; Beheshti-Maal et al., 2021).

The diversity and the composition of gut bacteria vary along the length of the gastrointestinal tract (GIT) with a gradual increase in bacterial load and diversity from the stomach and duodenum (10^2^ CFU/gm content) to the colon (10^11^–10^12^ CFU/gm content) (Eckburg et al., 2005; Eckburg and Relman, 2007; Frank et al., 2007; Sartor, 2008). The composition also varies at different intestinal locations starting with aerobic *Streptococcus* and *Lactobacillus* species in the duodenum and ending with strict anaerobes such as *Bacteroides*, *Bifidobacterium* and clostridial clusters in the colon (Frank et al., 2007). This difference in composition and diversity may be attributed to nutritional substrate availability, oxygen content, luminal acidity, and other physiological and immunological conditions at different parts of the GIT.

In addition to the stable differences in the composition at different intestinal locations, the gut bacteria are known to, significantly, vary among individuals (Eckburg et al., 2005). Furthermore, gut bacteria have been shown to be in continuous temporal variation within the same individual as revealed by monitoring the gut microbiota structure of two subjects for 15 months (Caporaso et al., 2011). These temporal variations could be attributed to small perturbations in some environmental factor, such as a short-term change in diet, a gastrointestinal infection, or due to a reversible change in the immunological state of the host (Caporaso et al., 2011; Lozupone et al., 2012). However, the gut microbiota tolerates these temporal changes and returns to its original structure once all physical and physiological conditions of the gut return to the normal state, which is known as resilience of the gut microbial ecosystem (Lozupone et al., 2012). Contrary to this, gut microbiota can lose this resilience by exposure to certain permanent factors, such as broad spectrum antibiotics (Lozupone et al., 2012), chronic diseases [e.g., Diabetes (Qin et al., 2012), IBD (Sartor, 2008)], obesity (Ray, 2012), or with age (Claesson et al., 2011). With regards to age, gut microbiota begins to colonize the intestine immediately after birth with a few aerobes (Enterobacteria, *Staphylococcus*, and *Streptococcus*), which gradually are replaced by anaerobic bacteria to reach the same complexity as mentioned above for adults by the first year of life (Palmer et al., 2007). However, differences between children and adults are still significant even after the first year of life. For example, children 1–7 years of age have lower fecal microbiota diversity with higher abundance of Enterobacteria than adults (Hopkins et al., 2001). Also, a more recent comprehensive study has indicated a substantial difference in elderly people when compared to the established adult pattern (Claesson et al., 2011), with Bacteroidetes as the predominant phyla and lower abundance of Firmicutes. This age-related pattern has been confirmed previously by comparative assessment of Firmicutes/Bacteroidetes ratio at different ages by quantitative PCR (Mariat et al., 2009). Also, individual bacterial species such as *Escherichia coli*, *Enterococci*spp., *Bacteroides*spp., *Bifidobacterium* spp., and lactobacilli have been demonstrated to exhibit specific age-related profiles in adults and elderly subjects (Woodmansey, 2007; Enck et al., 2009). Yatsunenko et al. (2012) linked the microbial diversity to both age and geographical location. They surveyed the gut microbiome structure in the stool of 314 Americans, 114 Malawians, and 100 Amerindians at different ages. They reported that the inter-subject variability is higher in the early stages of life compared to the adult microbiome with a progressive increase in microbial diversity with age (Yatsunenko et al., 2012). The authors also illustrated that the infant microbiome is dominated by Bifidobacteria and reaches an adult-like composition by the age of three (Yatsunenko et al., 2012). Moreover, the composition of the gut microbiota was more similar between Malawians and Amerindians and its diversity was higher than the American’s reflecting the association between geography and gut microbiota diversity.

## Role of Gut Microbiota in Human Health

Many reports have described humans as a superorganism that live in symbiosis with different microbes within various parts of the body. This ecosystem offers many benefits to the human host that are essential for good health. The gut harbors the greatest human microbial assembly (Sultan et al., 2020; El-Mowafy et al., 2021). The first role of the gut bacteria involves its metabolic capacity to process undigestible food particles such as complex carbohydrates, plant glycans, choline and bile acids (Tremaroli and Backhed, 2012). The microbial processing of indigestible polysaccharides generates beneficial short chain fatty acids (SCFAs) such as butyrate, acetate and propionate, which represent around 90% of SCFAs produced in the human gut (Cummings and Macfarlane, 1991). Butyrate is considered the major source of energy to the colonocytes. For example, the colonocytes of germ-free mice develop impaired mitochondrial respiration and increased autophagy in comparison with conventionally raised mice, and these findings have been reversed by adding butyrate to germ free colonocytes (Donohoe et al., 2011). On the other hand, acetate and propionate exert extra intestinal roles, where they act as metabolic substrates for lipogenesis and gluconeogenesis (Bergman, 1990; Tremaroli and Backhed, 2012). The major producers of SCFAs include the genus *Bacteroides*, Clostridium clusters IV and XIVa and *Bifidobacterium* (Cummings and Macfarlane, 1991; Louis et al., 2010; Martens et al., 2011). While *Eubacterium rectale*, *Roseburiafaecis*, *Eubacterium hallii*, and *Faecalibacterium prausnitzii* are the major butyrate producers in the gut as revealed by investigating the butyryl-CoA:acetate CoA-transferase gene (Louis et al., 2010), *B. thetaiotamicron* and *B. ovatus* showed a high genomic content of carbohydrate active enzymes (CAZymes) that enable them to metabolize indigestible plant and host glycans (Martens et al., 2011). This might explain the predominance of Bacteroidetes in the gut of rural Africans who have a mainly plant-based diet (De Filippo et al., 2010). The highest percentage of SCFAs in the large bowel is seen in the cecum and proximal colon, and decreases gradually toward the distal colon (Cummings and Macfarlane, 1991). This gradient may be explained by the higher prevalence of substrates in the proximal colon, which decreases progressively toward the rectum (Cummings and Macfarlane, 1991). The majority of the SCFAs produced are absorbed by the gut or delivered to peripheral tissues such as the liver and muscles (Bergman, 1990; Cummings and Macfarlane, 1991). Hence using feces to measure the gut metabolites may be inappropriate.

Gut microbiota, via various mechanisms, contributes to intestinal epithelial integrity. The first of these factors is the production of SCFAs, notably butyrate. Butyrate is a main modulator of mucin release, which acts as the first barrier against gut microbial invasion (Barcelo et al., 2000). In addition, butyrate controls gene expression of the colonocytes either via inhibition of histone deacetylase (HDAC) or through binding to G-protein coupled receptors (GPR41 or GPR43) (Tremaroli and Backhed, 2012). For example, sodium butyrate up-regulates the expression of the tight junction proteins and their mRNA via the inhibition of HDAC (Bordin et al., 2004). Furthermore, intra-rectal delivery of *C. tyrobutyricum* to immunocompetent and immunodeficient pathogen free mice showed a protective effect against dextran sulfate sodium (DSS)-induced colitis. This is mediated by inducing the expression of zonula occludens (ZO)-1 tight junction proteins, as well as MUC-2 mucin both of which are directly related to the luminal level of butyrate (Hudcovic et al., 2012). In addition to SCFAs, other microbial components contribute to the epithelial integrity. Germ free mice showed a thinner mucus layer relative to microbiota colonized mice, and this was corrected following exposure to LPS or peptidoglycan (Petersson et al., 2011). Recently, it has been shown that gut microbiota, also, induces intestinal mucosal endothelial and mesenchymal cells via stimulation of toll-like receptors (TLRs) and nucleotide-binding oligomerization domain (NOD) like receptor pathways (Schirbel et al., 2013). Together, this emphasizes the critical role of gut microbiota or their metabolites in maintaining the integrity of the intestinal barrier.

In addition to the development of tissues and cells, the gut microbiota is responsible for the shaping and maturation of the immune system. Germ free mice showed defective gut associated lymphoid tissues (GALT), fewer and smaller Peyer’s patches, less cellular mesenteric lymph nodes, less cellular lamina propria, lower expression of TLRs and class II major histocompatibility complex (MHC II) molecules, and finally reduced intraepithelial lymphocytes and CD4^+^ T cells in comparison to conventional mice. Additionally, gut colonization with microbes corrected some of these deficiencies (Lee and Mazmanian, 2010). In relation to specific pathogen free mice, germ free mice also showed accumulation of invariant natural killer T cells (iNKT) in the lamina propria of the colon and lung, which resulted in higher mortality rates (Olszak et al., 2012). However, the exposure of neonatal germ free mice, but not adults, to commensal bacteria was protective against iNKT-accumulation and its undesirable consequences (Olszak et al., 2012). Likewise, mice who received CD4^+^CD62L^+^ lymphocytes from germ free mice developed colitis faster than mice who received the same regulatory T cells (Tregs) from conventionally housed mice (Strauch et al., 2005). This suggests a critical role of the gut microbiota in the development of the intestinal immune system. This gut immune maturation is dependent on the host-specific microbiota. Colonization of germ free mice with either human or rat microbiota resulted in fewer intestinal T cells and dendritic cells, and lower antimicrobial peptide expression relative to germ free mice colonized with a murine microbiota, and humanized mice were more susceptible to salmonella infection (Chung et al., 2012). Both humanized mice and the mice colonized with a murine microbiota showed similar bacterial diversity at higher taxonomic levels, but they harbored different species (Chung et al., 2012). This indicates that each host selects a specific microbial consortium that shapes its immune system and maintains intestinal health.

## Gut Microbiota Dysbiosis in IBD

As mentioned above, our gut is populated with a complex and dynamic ecosystem, which under normal circumstances is characterized by a stable structure at various intestinal segments in everyone. Any alteration of this consortium may disrupt its functionality and eventually, a diseased state will appear. The dysbiosis of the intestinal microbiota is well reported in different diseases such as irritable bowel syndrome (IBS), obesity, diabetes, and IBD (Shanahan, 2007; Saulnier et al., 2011; Hansen et al., 2012; Qin et al., 2012; Everard et al., 2013). The relationship between IBD and gut microbiota dysbiosis was first established by studying animal models of colitis. Germ free IL 10^–/–^ mice do not develop colitis unless they are colonized by enteric bacteria (Schwerbrock et al., 2004). Garrett et al. (2007) were able to clearly demonstrate that the alteration of the gut microbiota composition could induce colitis in immunocompetent mice. They reported that deficiency of T-bet, a transcriptional factor that is important for gut homeostasis, resulted in microbial population shifts into a colitogenic community. This colitogenic microbiota was able to drive the intestinal inflammation in genetically intact mice (Garrett et al., 2007). Many molecular studies have illustrated the changes in gut microbial composition of IBD patients in comparison with non-IBD controls. The gut microbiota of individuals with IBD is characterized by low microbial diversity (Ott et al., 2004; Andoh et al., 2012), a reduced abundance of *Bifidobacterium* spp. (Joossens et al., 2011; Andoh et al., 2012), *Lactobacillus* spp. (Ott et al., 2004), and *F. prausnitzii* (Sokol et al., 2009; Joossens et al., 2011; Andoh et al., 2012) and a higher abundance of pathobionts such as adherent/invasive *E. coli* (Darfeuille-Michaud et al., 2004; Sokol et al., 2006a) and *C. difficile* (Rodemann et al., 2007), resulting in lower SCFA concentrations compared with healthy individuals (Huda-Faujan et al., 2010).

Studies using *16S rDNA* sequencing have shown a decrease in the diversity of gut biota in IBD mucosal specimens (Baumgart et al., 2007; Frank et al., 2007). Using single strand confirmation polymorphism (SSCP) fingerprint, based on *16S rRNA* showed that the diversity decreased by 50 and 30% in CD and UC, respectively (Ott et al., 2004). Frank et al. (2007) illustrated this imbalance by sequencing SS-rRNA clones from 190 biopsies. In their study, the *Lachnospiraceae* family of Firmicutes and Bacteroidetes were depleted in IBD subjects, with a relative increase of Proteobacteria, Actinobacteria, and Bacillus subgroups of Firmicutes. FISH analysis, on the other hand, has illustrated an increase in the relative abundance of Bacteroidetes and a low abundance of some butyrate producing bacteria such as *F. prausnitzii*in mucosal IBD specimens (Swidsinski et al., 2005; Sokol et al., 2008b, 2009). Using 454 pyrosequencing of the *16S rRNA* V5 and V6 regions extracted from the fecal materials of concordant and discordant twins, ileal CD, colonic CD and healthy individuals were differentiated from each other according to their microbial profile (Willing et al., 2010). However, the authors were not able to discriminate between UC and healthy subjects by following the same approach (Willing et al., 2010). Colonic CD was characterized by higher Firmicutes (mainly *Faecalibacterium*, and *Ruminococsulus*), *Bifidobacteriaceae* (*Bifidobacterium*), *Coriobacteriaceae* (*Collinsella*), and *Aneroplasmataceae*. Conversely, ileal CD showed depletion of *Ruminococaceae* family, especially *Faecalibacterium*, and *Collinsella* with higher abundance of Proteobacteria due to the increase of the *Enterobacteriaceae* family (Willing et al., 2010). For UC, Willing et al. (2010) were able to identify few differences such as depletion of *Prevotella*, *Streptococcus*, and *Asteroleplasma*. In contrast, other study was able to discriminate between UC and healthy individuals by calculating the mean of *16S rDNA*-clone libraries taken from sigmoid colon biopsies of 62 individuals (Lepage et al., 2011). The microbiota dysbiosis of UC was characterized by less diversity, fewer *Lachnospiraceae* and *Ruminococcacea* families with higher abundance of Proteobacteria and Actinobacteria (Lepage et al., 2011). These two studies confirm the association between IBD and gut microbiota dysbiosis. However, the discrepancy between them, regarding the UC microbiota structure, underlines the importance of the sampling approach in these types of studies (Mottawea et al., 2019).

For pediatric IBD, some studies have monitored the gut microbiota in IBD compared to healthy controls. Conte et al. (2006) described the alteration in microbiota composition along the length of the intestine in pediatric IBD patients in comparison to control subjects. They used the conventional culture-based techniques and *16S rRNA*-based real time PCR for quantifying the mucosa associated bacteria at the ileum, caecum, and rectum of 42 subjects. One important observation of this study is that, in contrast to adult IBD, *B. vulgatus* was found at lower abundance in patients with IBD compared to controls. This supports the idea that pediatric IBD is a unique form of IBD. Moreover, a study investigating the relative abundance of 9 bacterial groups using real-time PCR showed higher number of *E. coli* and lower number of *F. prausnitzii* in children with CD in comparison to control subjects (Schwiertz et al., 2010). Furthermore, microbiota diversity in pediatric IBD patients was shown to differ from that of adults (Cucchiara et al., 2009). However, these studies solely examined the dominant bacterial groups by applying conventional culture-based techniques and simple molecular methodology. Since it is widely accepted that 80% of gut microbiota are unculturable (Eckburg et al., 2005), a comprehensive molecular survey of the gut microbiota in pediatric IBD is necessary. Two studies have applied a high throughput molecular approach to characterize gut microbiota in pediatric IBD (Hansen et al., 2012; Papa et al., 2012). The first study applied synthetic learning in microbial ecology (SLiME) analytic approach of 454 pyrosequencing data obtained from fecal samples of 91 individuals and other published datasets. They were able to differentiate between children with IBD and healthy individuals or those with other diseases based on their microbiota composition (Papa et al., 2012). The drawback of this study is that they relayed upon stool samples for their analysis, and it is known that fecal materials contain different microbiota when compared to mucosa associated bacteria. Moreover, Hansen et al. (2012) reported that the microbiota diversity is lower in CD but not in UC relative to control subjects. Pairwise comparison among the 3 groups identified only 7 differentially abundant taxa. One important result is that *F. prausnitzii* was highly abundant in CD compared to controls, which is the reverse of what has been previously documented in adults with IBD (Sokol et al., 2008b; Hansen et al., 2012), indicating once again the unique microbiota composition of pediatric IBD. Using intestinal mucosal colonoscopic washes, Mottawea et al. (2016) have reported dysbiosis of gut microbiota in new onset pediatric IBD with enrichment of H_2_S producing bacteria. Overall, these studies support the association between intestinal microbiota imbalance and pediatric IBD at different ages. Nevertheless, the cause/effect relationship between these conditions and gut microbiota is still unclear.

Generally, microbiota dysbiosis is significantly greater in patients with CD than with UC (Pascal et al., 2017), where the microbial community stability and diversity is significantly lower in case of CD than in UC (Ryan et al., 2020). In case of CD a specific microbial signature that is comprised of eight groups is well reported. Eight groups of microorganisms involving *Anaerostipes*, *Methanobrevibacter*, *Faecalibacterium*, an unknown *Peptostreptococcaceae*, *Collinsella*, an unknown *Christensenellaceae*, and *Escherichia*, *Fusobacterium* could be utilized to differentiate between CD from non-CD, where, the first six groups are relatively low and the latest two groups are relatively high in case of CD (Pascal et al., 2017; Ryan et al., 2020). Moreover, it is noteworthy fecal (stool) and mucosal sampling is critical during the determination of the microbial dysbiosis in IBD (Lo Presti et al., 2019). The IBD stool samples are characterized by reduced diversity of microbiota in comparison to IBS and healthy population (Mei et al., 2021). IBD cases are characterized by decreased Verrucomicrobia and Bacteroidetes than healthy population (Lo Presti et al., 2019; Mei et al., 2021). On the other hand, in case of IBS Bacteroidetes are increased in comparison to healthy population. Moreover, IBD are reported to harbor less population of Bacteroidetes and Verrucomicrobia, and higher abundance of Actinobacteria in comparison to IBS (Lo Presti et al., 2019; Ryan et al., 2020; Mei et al., 2021). *Lactobacillus*, *Ruminococcus*, and *Streptococcus* are significantly higher in IBD than healthy population, while *Oscillospira*, *Lachnospiraceae*, *Ruminococcaceae*, and *Rikenellaceae*as well as *Akkermansia muciniphila* are diminished in IBD (Lo Presti et al., 2019; Ryan et al., 2020; Mei et al., 2021). In case of IBS, *Pseudomonas* and *Lactococcus* are decreased in comparison to healthy population, while *Parabacteroides distasonis*is relatively increased (Forbes et al., 2016; Lo Presti et al., 2019). By comparing IBS and IBD, *Rikenellaceae*, *Bacteroides*, *Butyricimonas*, *Oscillospira*, *Mogibacteriaceae*, *Anaerostipes*, *Barnesiellaceae*, *Roseburia*, *Parabacteroides*, *P. distasonis*, and *A. muciniphila* were more abundant in IBS than in IBD, while *Granulicatella* was relatively decreased (Dziarski et al., 2016; Lo Presti et al., 2019; Cuffaro et al., 2020). Similarly, to fecal samples, mucosal samples exhibited reduced microbiota diversity passing from healthy population to IBS to IBD (Lo Presti et al., 2019). Generally, there is no significant difference in microbiota population between inflamed and not-inflamed tissue samples of IBD. On contrary, the microbiota of inflamed mucosa of IBD patients exhibited low abundance of Firmicutes and Bacteroidetes and higher abundance of Proteobacteria in comparison to healthy population (Zuo and Ng, 2018). The abundance level of *Enterobacteriaceae* was significantly increased and the abundance levels of *Lachnospiraceae*, *Ruminococcaceae*, *Rikenellaceae*, *Bacteroides*, *Coprococcus*, *F. prausnitzii*, and *P. distasonis* were diminished in IBD inflamed mucosa compared to healthy population (Lo Presti et al., 2019; Aldars-García et al., 2021).

## Gut Mycobiome Dysbiosis in IBD

In addition to dysbiosis of bacteria, the dramatic changes that occur in the fungal community named as “mycobiome” is relatively important during IBD (Liu et al., 2020). Mechanistically, it appears that fungi may paly crucial role in the progression of IBD through either affecting the gut microbiota composition or increasing the production of pro-inflammatory cytokines (Iliev and Leonardi, 2017). In addition, mycobiome dysbiosis is a well-reported case in IBD (Beheshti-Maal et al., 2021). It is reported that fungal diversity is higher in CD patients than in healthy controls (Ott et al., 2008).

An increased ratio of Basidiomycota/Ascomycota is a characteristic feature of IBD (Sokol et al., 2017). Qiu et al. (2017) reported higher abundances of *Aspergillus*, *Wickerhamomyces*, *Candida*, and *Sterigmatomyces* and lower abundances of *Alternaria*, *Penicillium*, *Exophiala*, *Emericella*, *Acremonium*, *Epicoccum*, and *Trametes* in patients suffering from UC in comparison to healthy controls. However, there was no significant association between *Basidiomycota*/*Ascomycota* ratio and the increased levels of pro-inflammatory cytokines. Moreover, *Candida* spp., particularly *C. albicans*, is significantly increased in patients suffering from CD or general IBD (Li et al., 2014; Chehoud et al., 2015; Sokol et al., 2017). It is reported that specific-pathogen-free (SPF) Clec7a^–/–^ mice exhibit more severe colitis symptoms when colonized with the pathogenic fungus, *C. tropicalis* compared to uncolonized Clec7a^–/–^ mice or colonized wild type mice (Iliev et al., 2012; Tang et al., 2015). These studies confirm the crucial link between mycobiome dysbiosis, especially *Candida* spp. and IBD (Li et al., 2019). Li et al. (2014) illustrated higher levels of *Candida* spp. in the inflamed mucosa of IBD patients than in healthy population. In addition, Standaert-Vitse et al. (2009) illustrated higher colonization of familial CD patients by *C. albicans*. Moreover, other reports reported high levels of both *C. albicans* and *C. glabrata* in CD patients (Liguori et al., 2016; Sokol et al., 2017). In addition, Kowalska-Duplaga et al. (2019) documented the high abundance of *Candida* in CD patients, which was decreased by therapeutic intervention, especially with anti-TNF-α treatment.

Besides *Candida* spp., *Malasseziarestricta*, which is a skin normal fungus, significantly increases in patients suffering from CD (Limon et al., 2019). This fungus was found to aggravate the colitis in mouse models via mechanisms demanding on a protein included in antifungal immunity; CARD9 (Limon et al., 2019). Moreover, depletion of *Saccharomyces cerevisiae* is reported in IBD patients (Sokol et al., 2017). Tiago et al. (2015) depicted the protective effects of *S. cerevisiae* UFMG A-905 in mice suffering from UC. In addition, Sivignon et al. (2015) reported that adherent-invasive *E. coli* (AIEC)-induced ileal colitis can be reduced by *S. cerevisiae* CNCM I-3856 in a murine model. However, on the other hand, Chiaro et al., 2017 demonstrated that *S. cerevisiae* worsens the disease condition in a murine model of colitis (Chiaro et al., 2017).

The crosstalk between fungi and bacteria may be a pivotal concern in IBD patients. However, in pediatric IBD patients there was no significant correlation between leading fungal species with particular bacterial taxa and it has been hypothesized that fungal dysbiosis may be a result of or a cause for the gut bacterial dysbiosis (Chehoud et al., 2015). On the other hand, previous report showed a positive correlation between *C. tropicalis* and both *E. coli* and *Serratia marcescens* (Hoarau et al., 2016). It is noteworthy that the correlation between mycobiota-bacterial dysbiosis with the disease severity in IBD patients may afford evidence for the crucial role for gut mycobiome during IBD.

## Gut Virome Dysbiosis in IBD

In addition to microbiota and mycobiota, gut virome, which is comprised of viruses infecting both prokaryotes and eukaryotes, constitute a large portion of gut microbiome (Manrique et al., 2016; Lin and Lin, 2019). Bacteriophages are the major components of the enteric virome (Clooney et al., 2019). Generally, in patients suffering from IBD, alteration of the virome reflects microbiota dysbiosis (Clooney et al., 2019). One of the first studies, which reported virome dysbiosis in IBD patients revealed higher abundance of bacteriophages infecting *Alteromonadales*, *Clostridiales*, and *Clostridium acetobutylicum* as well as *Retroviridae* family in IBD patients in comparison to healthy population (Pérez-Brocal et al., 2015). It is well reported that Caudovirales phage families, including *Siphoviridae*, *Myoviridae*, and *Podoviridae*, are significantly enriched in IBD subjects (Wagner et al., 2013; Norman et al., 2015; Duerkop et al., 2018). A previous study illustrated the high abundance of Caudovirales phage in children suffering from IBD (Fernandes et al., 2019). On the other hand, another study reported a decreased diversity and consistency of Caudovirales that is directly connected to the inflammation degree of the intestine in UC patients (Zuo et al., 2019). Moreover, phages infecting *Enterobacteria* and *Escherichia* were reported to be abundant in UC patients (Zuo et al., 2019). A recent study established in germ-free mice showed that certain phages including *Escherichia*, *Lactobacillus*, and *Bacteroides* infecting phages as well as phage DNA exacerbate gut inflammation and contribute to IBD pathogenesis through increased production of IFN-γ via a TLR9-dependent pathway (Gogokhia et al., 2019).

In addition to bacteriophages, phages infecting and incorporating into eukaryotic cells are relatively important and have been related to IBD pathogenesis due to their ability of integration in human genome and affecting physiological state of the intestinal cells (Gloor et al., 2010; Beller and Matthijnssens, 2019; Santiago-Rodriguez and Hollister, 2019). Previous reports conducted on patients suffering from UC revealed higher abundance of *Pneumoviridae* and lower abundance of *Anelloviridae* compared to healthy population (Zuo et al., 2019). On the other hand, a study that was established on a small cohort of patients suffering from UC and CD depicted the higher abundance of *Herpesviridae* compared to healthy control (Wang et al., 2015). Other studies demonstrated that infection with Norovirus contributes to intestinal inflammation and can increase the rate of colitis incidence (Cadwell et al., 2010; Basic et al., 2014). Moreover, recent studies depicted the high abundance of *Hepeviridae* and *Hepadnaviridae* in the intestinal mucosa of patients suffering from CD and UC, respectively (Ungaro et al., 2019b). Despite all these studies, the actual role of virome dysbiosis during IBD has not been fulfilled yet, even with some reports revealing that intestinal inflammation may be initiated by eukaryotic viruses. By detecting and identifying viruses that infect IBD patients during the early stages of intestinal inflammation, it will be promising to establish a complete correlation between virome and disease progression.

**FIGURE 1:**
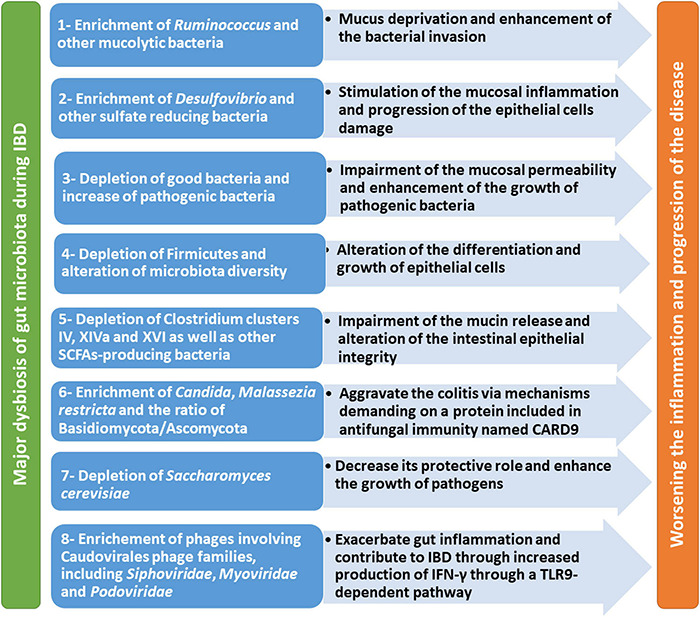
Major dysbiosis of gut microbiome during IBD and its impact on worsening the condition and progression of the disease.

Major dysbiosis of gut microbiota, mycobiome, and virome during IBD from different studies is summarized in Table 1. Moreover, the contribution of this dysbiosis to worsening the condition and progression of the disease is illustrated in Figure 1.

**TABLE 1:** Major dysbiosis of gut microbiome during IBD.

**Bacteriome dysbiosis**					

**Microorganism**	**Disease subtype**	**Dysbiosis**	**Model**	**Specimen**	**References**
Bifidobacteria, Firmicutes, *F. prausnitzii*	In both CD and UC	Decrease	Human	Mucosa	Sokol et al., 2008a
*Enterobacteriaceae* (AIEC, pathogenic *E. coli* B2+D group)		Increase			
Bifidobacteria, Lactobacilli		Decrease		Stool	
Enteroadherent *E. coli* associated with CD or UC		Increase			
Bacteroidetes, Cyanobacteria, *Bacteroides*, *Flavobacterium*, *Oscillospira*	In both CD and UC	Decrease	Human	Stool	Santoru et al., 2017
Firmicutes, Proteobacteria, Verrucomicrobia, Fusobacteria, *Escherichia*, *Faecalibacterium*, *Streptococcus*, *Sutterella*, *Veillonella*		Increase			
Proteobacteria (non-jejuni *Campylobacter*)	CD	Increase	Human	Stool or Mucosa	Zhang et al., 2014
Proteobacteria (*E. coli*)	CD	Increase	Human	Mucosa	Martin et al., 2004
*Yersinia enterocolitica*, *Bacteroides vulgatus*, *Helicobacter hepaticus*, Mycobacterial species	CD	Increase	Human	Stool	Khan et al., 2019
Firmicutes	In both CD and UC	Decrease	Human	Mucosa	Walker et al., 2011
Bacteroidetes		Increase			
*Enterobacteriaceae*	CD	Increase			
*Anaerostipes*, *Methanobrevibacter*, *Faecalibacterium*, an unknown *Peptostreptococcaceae*, *Collinsella*, an unknown *Christensenellaceae*	CD	Decrease	Human	Stool	Pascal et al., 2017
Escherichia, Fusobacterium		Increase			
Firmicutes, Bacteroidetes, and *Lachnospiraceae*	In both CD and UC	Decrease	Human	Mucosa	Frank et al., 2007
Proteobacteria and the Bacillus subgroup of Firmicutes		Increase			
Clostridium IXa and IV groups, *Bacteroides*, Bifidobacteria	In both CD and UC	Decrease	Human	Mucosa	Fava and Danese, 2011
Sulfate-reducing bacteria, *Escherichia coli*		Increase			
Enterobacteriaceae (*E. coli*)	CD	Increase	Human	Stool	Seksik et al., 2003
Bifidobacteria	CD	Decrease	Human	Stool	Favier et al., 1997
*Dialister invisus*, Clostridium cluster XIVa, *Faecalibacterium prausnitzii*, *Bifidobacterium adolescentis*	CD	Decrease	Human	Stool	Joossens et al., 2011
*Ruminococcus gnavus*		Increase			
*F. prausnitzii*	CD	Decrease	Human	Stool	Fujimoto et al., 2013
*Bacteroides* species, *Eubacterium* species, *Lactobacillus* species	In both CD and UC	Decrease	Human	Mucosa	Ott et al., 2004
Proteobacteria, *Enterobacteriaceae*		Increase			
Firmicutes	In both CD and UC	Decrease	Human	Stool or Mucosa	Morgan et al., 2012
Ruminococcus gnavus, Enterobacteriaceae: Escherichia/Shigella		Increase			
*Clostridium leptum* group (IV), and *Faecalibacterium prausnitzi*	In both CD and UC	Decrease	Human	Mucosa	Vrakas et al., 2017
*Bacteroides*		Increase			
*Bacteroides fragilis*	CD	Increase	Human	Stool	Walters et al., 2014
*Enterobacteriaceae*	In both CD and UC	Increase	Human	Stool	Jacobs et al., 2016
*Faecalibacterium* and *Roseburia*	CD	Decrease	Human	Stool	Willing et al., 2010
*Enterobacteriaceae*, *Ruminococcusgnavus*		Increase			
*Lactobacillus*, Bifidobacteria	In both CD and UC	Decrease	Human	Mucosa	Verma et al., 2010
*Bacteroides*, *Ruminococcus*		Increase			
*F. prausnitzii, Prevotella copri*	In both CD and UC	Decrease	Human	Stool	Halfvarson et al., 2017
*Enterobacteriaceae*, *Ruminococcus*		Increase			
*Roseburia* hominis, *Faecalibacterium prausnitzii*	UC	Decrease	Human	Stool	Machiels et al., 2014
Firmicutes, Erysipelotrichales, Bacteroidales, Clostridiales	CD	Decrease	Human	Mucosa	Gevers et al., 2014
*Enterobacteriaceae*, *Pasteurellacaea*, *Veillonellaceae*, *Fusobacteriaceae*		Increase			
Firmicutes, *F. prausnitzii*, Bacteroidetes	In both CD and UC	Decrease	Human	Mucosa	Tong et al., 2013
Proteobacteria		Increase			
*F. prausnitzii*	In both CD and UC	Decrease	Human	Stool or mucosa	Wang et al., 2014
Bifidobacteria, *Lactobacillus* group		Increase			
*Enterobacteriaceae*	CD	Increase	Human	Stool	Seksik et al., 2003
Firmicutes	TNBS colitis	Decrease	Animal (rats and mice)	Mucosa	Ettreiki et al., 2012
Bacteroidetes, *Bacteroides*, *Enterobacteriaceae*		Increase			
*Helicobacteraceae*, *Mucispirillum, Desulfovibrio*	Experimental colitis	Increase	T-bet(−/−), Rag2(−/−) mice	Mucosa	Rooks et al., 2014
*Enterobacteriaceae* and AIEC	Experimental colitis	Increase	IL-10(−/−) mice	Mucosa	Arthur et al., 2012
*Akkermansia muciniphila*, *Bacteroides distasonis*, *Enterobacteriaceae*, *Clostridium ramnosum*	DSS-colitis	Increase	Animal (mice)	Mucosa	Håkansson et al., 2015
*Porphyromonas* genera, *Bacteroides*	Experimental colitis	Increase	APC(Δ468) IL-10(−/−) mice	Mucosa	Dennis et al., 2013

**Mycobiomedysbiosis**					

**Microorganism**	**Disease**	**Dysbiosis**	**Model**	**Specimen**	**References**

*Alternaria*, *Penicillium*, *Exophiala*, *Emericella*, *Acremonium*, *Epicoccum*, and *Trametes*	UC	Decrease	Human	Mucosa	Qiu et al., 2017
*Aspergillus*, *Wickerhamomyces*, *Candida*, and *Sterigmatomyces*		Increase			
*Candida* spp., particularly *C. albicans*	CD or general IBD	Increase	Human	Stool or Mucosa	Li et al., 2014
Basidiomycota/Ascomycota ratio, *Candida* spp., particularly *C. albicans*	In both CD and UC	Increase	Human	Stool	Sokol et al., 2017
*Candida* spp., particularly *C. albicans*	CD	Increase	Human	Stool	Standaert-Vitse et al., 2009; Sokol et al., 2017; Kowalska-Duplaga et al., 2019
*Candida* spp. particularly C. glabrata	CD	Increase	Human	Mucosa	Liguori et al., 2016
*Candida* spp. *C. albicans*, *C. tropicalis*	Experimental colitis	Increase	Clec7a^–/–^ mice	Mucosa	Dalmasso et al., 2006; Rodríguez-Nogales et al., 2018

**Virome dysbiosis**					

**Microorganism**	**Disease**	**Dysbiosis**	**Model**	**Specimen**	**References**

*Malasseziarestricta*	CD	Increase	Human and animal (mice)	Mucosa	Zheng et al., 2015
Bacteriophages infecting Alteromonadales, Clostridiales, and *Clostridium acetobutylicum* as well as Retroviridae family	CD	Increase	Human	Stool or Mucosa	Pérez-Brocal et al., 2015
Caudovirales phage families, including *Siphoviridae*, *Myoviridae*, and *Podoviridae*	Experimental colitis	Increase	Animal (mice)	Mucosa	Galtier et al., 2017
Caudovirales phage	In both CD and UC	Increase	Human	Stool	Fernandes et al., 2019
Caudovirales phage, Anelloviridae	UC	Decrease	Human	Stool	Zuo et al., 2019
Enterobacteria and *Escherichia* infecting phages, Pneumoviridae		Increase			
*Escherichia*, *Lactobacillus*, and *Bacteroides* infecting phages as well as phage DNA	Experimental colitis	Increase	Animal (mice)	Mucosa	Virgin, 2014
Caudovirales phage families, including *Siphoviridae*, *Myoviridae*, and *Podoviridae*	In both CD and UC	Increase	Human	Stool	Lim et al., 2015
Caudovirales phage	CD	Increase	Human	Mucosa	Kernbauer et al., 2014
Herpesviridae	In both CD and UC	Increase	Human	Mucosa	Wang et al., 2015
Norovirus	Experimental coltits	Increase	Animal (mice)	Mucosa	Basic et al., 2014
Herpesviridae	CD	Increase	Human	Mucosa	Ungaro et al., 2019b
Hepadnaviridae	UC	Increase	Human	Mucosa	Ungaro et al., 2019b

## Dysfunctionality of Gut Microbiota in IBD

Two metagenomic studies tried to link the dysfunctionality of the gut microbiome and IBD via two different approaches (Greenblum et al., 2012; Morgan et al., 2012). First, Greenblum et al. (2012) treated the gut microbiota as one single organism and constructed the metabolic network of the entire community and compared the odd ratios, of the topology of different metabolic pathways, genes and biological processes of obese and IBD cases against healthy individuals. This approach yielded an association between IBD and genes involved in NO_2_ production and metabolism of choline and p-cresol (Greenblum et al., 2012). In contrast to the metagenomic analysis of the community as a single supra-organism, Morgan et al. (2012) identified the microbiota composition of each disease phenotype. Then, they extracted the genome of the microbiota composing each community and constructed a gene catalog for each phenotype, followed by genes and pathway assessment using sparse multivariate analysis (Morgan et al., 2012). They also confirmed their findings by shotgun metagenomic analysis of 4 CD and 7 healthy fecal microbiota (Morgan et al., 2012). They found an IBD-associated enrichment of genes involved in glutathione transport and metabolism, sulfur amino acid metabolism, redox homeostasis, mucin degradation, secretion systems, adhesion, and invasion as well as a depletion of genes involved in the biosynthesis of SCFAs, some nucleotides and amino acids (Morgan et al., 2012). Recently, an interesting study identified a network of bacterial metabolite interactions including sulfur metabolism as an important player correlated to CD activity (Metwaly et al., 2020). To better understand the dysfunctionality of gut microbiota in IBD, we will discuss the IBD-associated metabolic shift in more detail.

### Hydrogen Sulfide Metabolism

Gut microbiota generate hydrogen sulfide (H_2_S) via 2 different biochemical pathways. First, sulfate reducing bacteria (SRB) couple the reduction of sulfate as a terminal electron acceptor to the oxidation of H_2_ or other organic compounds such as lactate as an electron donor during their anaerobic respiration (Carbonero et al., 2012). The second pathway is followed by bacteria that ferment sulfur containing amino acids. These bacteria might depend on desulfhydrases such as cysteine desulfhydrase or other enzymes, which have the ability to metabolize cysteine (Awano et al., 2005). Sulfate reducing bacteria mainly include members of Deltaproteobacteria in addition to *Desulfotomaculum*, *Desulfosporosinus*, *Thermodesulfobacterium*, and *Thermodesulfovibrio* genera (Blachier et al., 2010). Using multiplex PCR to identify fecal-SRB isolates, luminal SRB are dominated by *Desulfovibriopiger*, *D. fairfieldensis*, and *D. desulfuricans* (Loubinoux et al., 2002). A diversity analysis of *Desulfovibrio* using the dissimilatory sulfite reductase (*dsrAB*) gene confirmed the predominance of *D. piger* with the detection of a new unclassified SRB (Scanlan et al., 2009). Another study identified 8 sulfate and sulphite reducing bacteria in addition to the sulphite reducing bacterium, *Bilophilawadsworthia* via 454 pyrosequencing of *dsrAB* gene fragment (Jia et al., 2012). The identified bacteria include four known species; *D. piger*, *D. vulgaris*, *Desulfovibrio* sp. *NY682*, and *D. desulfuricans F28-1*, and four new species highly similar to *D. desulfuricans* F28-1 (93% *dsrAB*sequence similarity); *D. oxamicus* (84% identity), *Desulfotomaculum* sp. *Lac2* (80% identity), and *D. simplex* (88% identity) (Jia et al., 2012). H_2_S producing microbiota through fermentation of amino acids include, for example, *Fusobacterium nucleatum*, Atopobium spp., *Gemella sanguinis*, *Micromonas micros*, *Streptococcus* spp., *Actinomyces* spp., *Eubacterium* spp., *Veillonella* spp., *Bulleidiamoorei.*, *Prevotella* spp., *Campylobacter* spp., and *Selenomonas* spp. (Washio et al., 2005). Mottawea et al. (2016) have reported the enrichment of H_2_S producers in children with CD with one H_2_S generating organism; *A. parvulum*, has proinflammatory characteristics in IL10^–/–^ mice. Recently, an interesting study identified a network of bacterial metabolite interactions including sulfur metabolism as an important player correlated to CD activity (Metwaly et al., 2020). The authors showed that CD patients with active disease are enriched in members belonging to *Enterococcus*, *Fusobacterium*, *Haemophilus*, *Megasphaera*, and *Campylobacter*, while *Roseburia*, *Christensenellaceae*, *Oscillibacter*, and *Odoribacter* are enriched in CD patients with inactive disease (Metwaly et al., 2020). Another report detected two dominating sulfate-reducing bacteria morphotypes that differ in colonial size and quantitate in the feces of healthy and patients with colitis. In the feces of healthy individuals, 93% of sulfate-reducing bacteria of morphotype I prevailed (*Desulfovibrio*) while morphotype II included only 7% (*Desulfomicrobium*); in the feces of patients with colitis, the ratio of these morphotypes was 99:1, respectively (Kushkevych et al., 2021). In addition to microbiota-released H_2_S, it can also be synthesized endogenously by intestinal colonocytes from L-cysteine via two main enzymes; cystathionine β-synthase and cystathionine γ-lyase (Shibuya et al., 2013). Two additional pathways (3-mercaptopyruvate sulfurtransferase in combination with cysteine aminotransferase and 3-mercaptopyruvate sulfurtransferase coupled with D-amino acid oxidase) were identified in H_2_S production, peripherally (Shibuya et al., 2013; Guo et al., 2016). Normally, the luminal H_2_S concentration of the human large intestine is 1.0–2.4 mmol/L (Macfarlane et al., 1992), while the concentration in the fecal contents ranges from 0.17 to 3.38 mmol/kg (Florin, 1991; Magee et al., 2000). Taking into consideration the lipid solubility and the passive diffusion of H_2_S through the intestinal mucosa, these concentrations are underestimated.

At lower concentrations (<1 mM), H_2_S is considered a cytoprotective metabolite that induces some cellular anti-inflammatory responses. These cellular responses include, for example, prevention of caspase activation and apoptotic cell death (Rose et al., 2005), inhibition of leukocyte adhesion to vascular endothelium, which decreases infiltration of neutrophils and lymphocytes (Zanardo et al., 2006), induction of cyclooxygenase-2 (COX-2) expression (Wallace et al., 2009), and promotion of neutrophil apoptosis (Mariggio et al., 1998). The later was contradicted by Rinaldi et al. (2006) who concluded that H_2_S accelerates the resolution of the inflammation process via inhibition of polymorphonucelar (PMN) apoptosis. In another *in vitro* study, H_2_S at normal colonic concentration lowered the proliferation of different colonic cancerous and normal cells and induced autophagy through the AMP-activated protein kinase (AMPK) pathway (Wu et al., 2012). At higher concentrations, H_2_S acts as a genotoxic and/or cytotoxic transmitter to the colonocytes by affecting genes responsible for cell cycle progression, DNA repair and inflammatory responses (Attene-Ramos et al., 2010). The main cytotoxic effect of H_2_S is the inhibition of cytochrome c oxidase activity, which is the terminal oxidase of mitochondrial respiration (Roediger et al., 1993; Leschelle et al., 2005). This leads to the prevention of the oxidation of essential metabolites such as n-butyrate, L-glutamine and acetate, which eventually decreases the bioenergetic performance of the cell (Roediger et al., 1993; Leschelle et al., 2005). Sulfide has also been shown to inhibit butyrate oxidation in rat colonocytes through inhibition of short chain acyl dehydrogenation of activated fatty acids (Moore et al., 1997). The antagonistic effect of H_2_S on butyrate may induce hyperproliferation of the colonic mucosa (Christl et al., 1996). An indirect cytotoxicity of H_2_S was reported, where increased sulfide production induces the conversion of nitrite to nitric oxide, which had a damaging effect on colonocytes (Vermeiren et al., 2012).

To keep the local concentration of H_2_S at a harmless level, the colonic mucosa expresses a special H_2_S oxidation system that degrades H_2_S to sulfate and thiosulfate (Furne et al., 2001). This oxidation system consists of sulfide quinone reductase (SQR), dioxygenase ethylmalonic encephalopathy protein 1 (ETHE1), and thiosulfate sulfur transferase (TST that is also known as rhodanese) (Mimoun et al., 2012). This mitochondrial oxidation of H_2_S is not essential for cell respiration, but instead, its main purpose is the detoxification of excess H_2_S (Mimoun et al., 2012). In contrast, the respiratory capacity of the cell is an important parameter that affects the efficiency of H_2_S detoxification independent of the mitochondrial oxidation system (Mimoun et al., 2012).

A higher abundance of H_2_S generated by gut microbiota is considered one of the strongest models associated with the pathogenesis of IBD. Higher luminal H_2_S concentration in IBD could arise from either increased abundance of H_2_S-producing bacteria or a deficient H_2_S-detoxification pathway. The association between H_2_S and IBD was first reported in UC. In Roediger et al. (1997) reviewed the role of H_2_S in the pathogenesis of UC. They stated that the level of colonic sulfide and the relative abundance of sulfate reducing bacteria was higher in UC patients compared to healthy subjects (Roediger et al., 1997). In addition, the bacteria isolated from UC patients showed higher generation of H_2_S than those separated from control cases (Roediger et al., 1997). Treatment of those patients with 5-aminosalicylic acid containing drugs lowered the production of H_2_S as indicated by the stool sulfide level and this was proposed to contribute to the therapeutic activities of these drugs (Edmond et al., 2003). Sulfate reducing bacteria were exclusive to patients with UC, where they were isolated from 80% of UC pouches but not form patients with familial adenomatous polyposis (Duffy et al., 2002). Other indirect evidences for the role of H_2_S in the pathogenesis of UC are also available. The first comes from studies of diet consumption, where high protein intake is associated with a higher risk of IBD (Ng et al., 2013). Higher protein means increased sulfur containing amino acids and subsequently, elevated H_2_S levels. The second is the higher activity of fecal mucin sulphatase in UC patients (Tsai et al., 1995). Mucin sulphatase releases sulfate from the mucosal sulfomucin and this endogenous sulfate provides the source for H_2_S biosynthesis by SRB (Tsai et al., 1995). Regarding CD, few reports have linked H_2_S production with disease activity. Jia et al. (2012) have reported no difference in the general abundance of SRB between CD and healthy subjects. Mottawea et al. (2016) have illustrated the increased abundance of H2S producing bacteria along with downregulation of mitochondrial proteins implicated in H2S detoxification in children with CD. Some indirect links are also available. For example, increased metabolism of sulfur containing amino acids such as methionine and cysteine associated with a decrease in the metabolism of sulfur lacking amino acids such as lysine and glutamine are characteristics of ileal CD (Morgan et al., 2012). Also, the same study reported an over representation of sulfate transport genes in CD patients. Both sulfate and sulfur containing amino acids are the main precursors for H_2_S biosynthesis as mentioned above. The second factor that contributes to higher colonic sulfide concentration is the dysfunctionality of H_2_S detoxification. The dysfunctionality of H_2_S detoxification genes in IBD is still up for debate. Pitcher et al. (1998) showed that the activity of thiol-methyl transferase (TMT) is higher in the peripheral blood of UC patients. This was then confirmed in 2007, when the activity of TMT and rhodanese were found to be higher in the erythrocytes of UC patients than controls (Picton et al., 2007). However, no difference in TMT and rhodanese activity was detected in the rectal biopsies of the same individuals. In patients with CD neither the erythrocytes nor the rectal biopsies of patients showed a change in that enzymatic activity (Picton et al., 2007). In 2009, the role of H_2_S detoxification in IBD emerged again when the activity and the expression of rhodanese were shown to decrease in parallel to the development of dextran sodium sulfate-induced colitis in mice (Taniguchi et al., 2009). In concordance with formerly mentioned studies, this loss of activity is followed by an increase in its activity in red blood cells (Taniguchi et al., 2009). Impaired detoxification of H_2_S has been confirmed for UC patients via the assessment of TST expression level and activity in colonic mucosal biopsies (De Preter et al., 2012). As well, it has been confirmed for CD where metaproteomic and expression analyses reported the decreased abundance of H2S detoxification proteins and transcripts in children with new onset CD and UC (Mottawea et al., 2016). These includes the sulfur dioxygenase (*ETHE1*), the thiosulfate sulfurtransferase (*TST*) and the components of complexes III and IV of the mitochondrial respiratory chain, and *tst*, cytochrome *c* oxidase subunit IV (*hcox41*) and the sulfide dehydrogenase genes (*SQRDL*) transcripts (Mottawea et al., 2016). All in all, the association between higher H_2_S generation and IBD is well established. Additionally, the impaired intestinal H_2_S detoxification in IBD is evident.

### Short Chain Fatty Acids Metabolism

Butyrate is known as the salt or ester of butanoic (butyric) acid, which is a weak acid with a pKa of 4.8. By considering the pH of the intestine, which is approximately neutral, most of the butyrate in the intestine will be in the anionic form rather than the free acid form. Butyrate biosynthesis by the gut microbiota starts by condensation of two molecules of acetyl coA to generate one molecule of butyrylcoA. Next, butyrate are generated from butyrylcoA via two main pathways; the enzymes butyrate kinase and phosphotransbutyrylase or butyryl-CoA: acetate-CoA-transferase (Louis and Flint, 2009). The second pathway has been shown to be predominant among butyrate producing microbiota in human colon (Duncan et al., 2002). The major butyrate producing bacteria that inhabit the human gut are related to Clostridium clusters XIVa and IV with few percentage of Clostridium clusters I, XV, and XVI (Louis and Flint, 2009), while acetate and propionate are mainly produced by *Bacteroides* (Martens et al., 2011). It was indicated that the terminal ileum and proximal colon are the main sites of butyrate production, while acetate and propionate are generated in the distal colon, where *Bacteroides*is the predominant bacteria (Walker et al., 2005). Particularly, the role of butyrate in preventing IBD is illustrated in several studies as mentioned below.

A large body of evidence has revealed the association between IBD, perturbations of butyrate metabolism and/or depleted butyrate producing bacteria in the gut. Initially, a raised luminal butyrate level, as a result of impaired oxidation by colonocytes, was correlated with the severity of mucosal inflammation and was considered as a biomarker in UC patients (Roediger, 1980; Roediger et al., 1982). On the other hand, butyrate intake was shown to have a protective effect against development of colitis independent of restored butyrate oxidation in UC patients (De Preter et al., 2011). For example, administration of butyrate either orally or via local enema to mice or rats with chemically induced colitis alleviated the mucosal inflammation (Butzner et al., 1996; Vieira et al., 2012). Indeed, butyrate exhibits several anti-inflammatory activities. First, butyrate inhibits NFκB activation, which results in suppression of proinflammatory cytokines in UC patients (Luhrs et al., 2002) and rats with trinitrobenzene sulphonic acid (TNBS) induced colitis (Segain et al., 2000). This inhibitory action is impaired in IBD individuals as revealed by assessment of butyrate effect on cytokines production by peripheral blood mononuclear cells (PBMC) isolated from IBD and healthy subjects in response to TLR-2 activation (Kovarik et al., 2011). Secondly, butyrate was reported to induce Fas-mediated apoptosis of T cells via inhibition of HDAC-1 in mice (Zimmerman et al., 2012). This in turn inhibits IFN-γ-induced STAT1 activation, which results in reduced colonocyte expression of inflammatory mediators such as nitric oxide synthase (iNOS) and cyclooxygenase 2 (COX2) (Zimmerman et al., 2012). Finally, Butyrate is known to reduce the inflammation through contributions to intestinal barrier integrity. For example, intra-rectal inoculation of *C. tyrobutyricum*, the potent butyrate producer, has restored MUC-2 secretion, upregulated expression of (ZO)-1 tight junction protein and reduced cytokine release in DSS-treated mice (Hudcovic et al., 2012). Recently, Magnusson et al. (2020) compared impacts of butyrate on the intestinal immune profile of UC patients with active disease and non-inflamed controls. They found that butyrate exhibits different impact on gene regulation and more strongly leads to down-regulation of +expressed genes of inflammatory pathways in non-inflamed controls than in inflamed tissue of UC patients (Magnusson et al., 2020). According to the authors, such discrepancies can partly elucidate why expected anti-inflammatory impacts of local butyrate stimulation or supplementation are not always obtained.

Regarding butyrate producing bacteria, screening of fecal microbiota of 6 healthy and 6 CD individuals via *16S rDNA* clone sequencing and FISH analysis revealed a depletion of *C. leptum* cluster in CD cases compared to the healthy group (Manichanh et al., 2006). In agreement with this, Sokol et al. (2008b) illustrated that *F. prausnitzii* was depleted in CD patients mucosal microbiota and this reduction in abundance was associated with a higher risk of recurrence of ileal CD. Though, they have shown that the anti-inflammatory activity of this bacterium is independent of butyrate production using *in vitro* cellular models and *in vivo* colitis model of mice. Recently, depletion of *F. prausnitzii*and other butyrate producers was confirmed in the fecal microbiota of adults with CD compared to their unaffected relatives and other healthy controls via denaturing gradient gel electrophoresis and real time PCR (Joossens et al., 2011). This is not the case in pediatric CD, where some studies reported that *F. prausnitzii* had higher abundance in CD in comparison to controls (Hansen et al., 2012; Mottawea et al., 2016). An important difference in the pediatric studies is that the samples were collected at the time of diagnosis by colonoscopy, in contrast to Sokol et al. (2008b) where specimens were taken at surgical resection. This indicates the importance of sample collection time for these kinds of microbiota studies. The age groups screened by different studies were also different confirming the different phenotypes of the disease at different ages.

Geirnaert et al. (2017) reported the therapeutic effect of butyrate-producing bacteria in CD patients in addition to its role in enhanced intestinal epithelial barrier integrity. The four-strain probiotic supplement (containing *Lactobacillus acidophilus* NCIMB 30175, *Lactobacillus plantarum* NCIMB 30173, *Lactobacillus rhamnosus* NCIMB 30174, and *Enterococcus faecium* NCIMB 30176) was found to positively influence the immune response via colonic butyrate production *in vitro* and facilitating modulation of the gut microbiota composition and metabolic enhancing (Bjarnason et al., 2019; Moens et al., 2019).

### Bile Acid Metabolism

Bile acids are self signaling molecules that regulate their own biosynthesis through a negative feedback mechanism (Sayin et al., 2013). Primary bile acids are the product of cholesterol breakdown in the liver, while secondary bile acids are the products of gut microbiota metabolism. primary and secondary bile acids have been found to act as signaling players on a group of cell membrane and nuclear receptors together named “bile acid-activated receptors.” These receptors are highly expressed all over the gastrointestinal tract and control the bilateral communications of the intestinal microbiota with the host immune system (Fiorucci et al., 2021). Bile acid absorption is affected in models of inflammatory bowel disease (Fitzpatrick and Jenabzadeh, 2020). The expression of bile acid transporter apical sodium dependent bile acid transporter was inhibited in rats with colitis in addition to murine, canine and rabbit models of intestinal inflammation (Fitzpatrick and Jenabzadeh, 2020).

Bile acids mainly act as digestive aids to facilitate digestion of cholesterol, fat-soluble vitamins and triglycerides into water soluble products so they can be absorbed from the small intestine (Russell, 2003). They are also nutrient regulatory molecules that mediate some endocrine functions (Houten et al., 2006; Hylemon et al., 2009). They activate some nuclear receptors and cell signaling pathways to regulate lipid and glucose metabolism, energy expenditure and triglyceride homeostasis (Watanabe et al., 2006; Hylemon et al., 2009; Trauner et al., 2010). In addition, they have been shown to exert antimicrobial activity either directly through their detergent characteristics on bacterial membranes or via indirect physiological function (Hofmann and Eckmann, 2006; Duboc et al., 2013). Their indirect antimicrobial activity mainly occurs in the distal small intestine, where they promote the activation of nuclear farnesoid X receptor (FXR) (Inagaki et al., 2006). Activation of FXR induces the expression of several host genes involved in mucosal defense including genes involved in oxidative stress, and antibacterial peptide biosynthesis (Bernstein et al., 1999; Hofmann and Eckmann, 2006; Inagaki et al., 2006). The expression and function of bile acid-activated receptors including FXR in addition to other receptors; G-Protein bile acid-activated receptor, pregnane-X-receptor, vitamin D receptor, and related orphan receptor gamma are strongly linked to the composition of the intestinal microbiota and negatively regulated by intestinal inflammation (Fiorucci et al., 2021).

The enterohepatic circulation of bile acids starts at the liver, where 14 enzymes are required to synthesize the two primary forms of bile acids namely cholic acid and chenodeoxycholic acid from cholesterol (Russell, 2003). Thereafter, they are conjugated to glycine or taurine to form bile salts (also termed conjugated bile acids), which are stored in the gallbladder during interdigestive periods. After meals, bile salts are released into the duodenum via the biliary duct to facilitate lipid digestion and absorption (Ridlon et al., 2006). In the gut lumen, the microbiota induce biotransformation of the primary forms of bile acids into secondary bile acids products via deconjugation, oxidation of hydroxyl groups and dehydoroxylation (Ridlon et al., 2006). This increases the hydrophobicity of bile acids so that they can passively diffuse through the small intestine and proximal colon in addition to their active transport from the small intestine to the blood stream and back to the liver (Ridlon et al., 2006). Approximately 95% of bile acids are recycled and transported through the circulation to return to the liver while 5% (400–600 mg) enter the colon, where they are dehydroxylated by colonic bacteria into secondary bile acid products [Deoxycholic acid (DCA) and Lithocholic acid (LCA); Ridlon and Hylemon, 2012].

Gut microbiota start the metabolism of bile salts via the deconjugation step through bile salt hydrolases (BSH) that generate free primary bile acids and amino acids (Ridlon et al., 2006). The distribution of BSH activity among gut microbiota was estimated via metagenomic analysis of 89,856 fecal clones (Jones et al., 2008). Firmicutes were the most predominant BSH-expressing bacteria (30%) followed by Bacteroidetes (14.4%) and Actinobacteria (8.9%) (Jones et al., 2008). Firmicutes with BSH activity include the genera *Eubacterium*, *Coprococcus*, *Clostridium*, *Ruminococcus*, *Dorea*, *Lactobacillus*, *Enterococcus*, *Listeria*, and *Lactococcus* while *Bifidobacterium* and *Collinsella* are the two Actinobacteria genera that express BSH (Jones et al., 2008). The same study revealed that Firmicutes and Actinobacteria are capable of deconjugating both glyco- and tauro-conjugated bile acids while Bacteroidetes are only able to hydrolyse tauro-conjugated bile acids. Also 9 BSH open reading frames have been identified among the gut microbiota community, one of them shares 56% homology with a protein encoded by the gut, archeon *Methanobrevibacter smithii*, which has been confirmed to have BSH activity against both glyco- and tauro-conjugated bile acids (Jones et al., 2008). The second step of bile acid metabolism by intestinal bacteria is the oxidation of the hydroxyl group at carbon 3, 7, or 12 of deconjugated bile acids via hydroxysteroid dehydrogenases to generate the oxo-derivative of bile acids (Ridlon et al., 2006). Gut microbiota expressing hydroxysteroid dehydrogenases include, but are not limited to *Clostridium* spp., *Eggerthella lentum*, *Ruminococcus* spp., *Bacteroides* spp., and *E. coli* (Ridlon et al., 2006; Fukiya et al., 2009). Simultaneously, some microbes promote 7α-/β-dehydroxylation of free cholic acid and chenodeoxycholic acid to generate deoxycholic acid (DCA) and lithocholic acid (LCA), respectively (Ridlon and Hylemon, 2012; Kakiyama et al., 2013). This dehydroxylation activity is common among the order Clostridiales of gut bacteria including *Ruminococcaceae*, *Lachnospiraceae*, and *Blautia* (Kakiyama et al., 2013). Heinken et al. (2019) performed an interesting study that included a systematic workflow to computationally model bile acid metabolism by gut microbes and microbial communities. The authors found that, each microbe could produce maximally 6 secondary bile acids *in silico*, while microbial pairs could produce up to 12 bile acids, suggesting bile acid biotransformation being a microbial community task (Heinken et al., 2019). Das et al. (2019) performed a shotgun metagenomic analysis of the bile salt biotransformation genes and their distribution at the phyla level. They reported that IBD patients harbored a significantly lower abundance of these genes in comparison with healthy individuals; many of these genes originated from Firmicutes (Das et al., 2019).

Bile acids metabolism by gut microbes has a controversial contribution to both the host and the microbe. The deconjugation step has been suggested to benefit gut microbiota colonization by increasing their resistance to bile salts, where the antimicrobial effect of bile acids benefit the host via alleviating the bacterial overgrowth (Desmet et al., 1995; Ridlon et al., 2006; Jones et al., 2008). Also, bile acid modification results in reducing cholesterol level and controls lipid metabolism, which is considered a protective factor against metabolic disorders such as obesity, cardiac diseases and diabetes (Tonack et al., 2013; Wu et al., 2013). Another link between bile acids and metabolic diseases arises from its effect on gut microbiota composition. Using *16S rDNA* clones sequencing of rats’ cecal microbiota has revealed that cholic acid is a host modifier of the gut microbiota structure that is equivalent to high fat diet (Islam et al., 2011). One more benefit to the host from bile acid metabolism is the protection from pathogen colonization such as vegetative *C. difficile* (Sorg and Sonenshein, 2008). However, high levels of secondary bile acids are associated with some diseases such as gastrointestinal cancer and gallstones (Berr et al., 1996; Bernstein et al., 2005).

Four decades of research have established the association between IBD and bile acid dysmetabolism (Andersson et al., 1978; Heuman et al., 1983; Nyhlin et al., 1994; Duboc et al., 2013; Iwamoto et al., 2013). Different studies have linked the dysmetabolism of bile acids to gut microbiota dysbiosis in IBD (Wohlgemuth et al., 2011; Duboc et al., 2013). It is clear now that cholic acid or a high fat diet act as a regulatory host factor that selects for gut microbes that have the machinery to detoxify bile acid metabolites or utilize their conjugates, glycine and taurine as metabolic substrates (De Filippo et al., 2010; Islam et al., 2011; Devkota et al., 2012). Devkota et al. (2012) reported that diets high in saturated fat induce the expansion of the H_2_S producing pathobiont, *Bilophila wadsworthia*, which in turn promotes colitis in IL10^–/–^ mice. This proinflammatory effect of high saturated fat diet was attributed to taurocholic acid (Devkota et al., 2012). Taurine, the conjugate in taurocholic acid, is known to have a sulfonic acid moiety that could be dissimilated by gut microbiota into H_2_S as a microbial by-product (Laue et al., 2001). Diets high in fat or meat lead to more conjugation of taurine to bile acids and consequently more H_2_S production, which is considered as a potential risk factor for IBD (Magee et al., 2000; Devkota et al., 2012). Alternatively, microbial dysbiosis associated with IBD may result in bile acid dysmetabolism and this in turn may affect the anti-inflammatory characteristics of bile acids (Duboc et al., 2013). Duboc et al. (2013) has reported the dysfunctionality of bacterial metabolism of bile acids because of microbial dysbiosis. Moreover, they illustrated that this intestinal dysmetabolism disturbed the intestinal bile acid pool, which in turn impacted the anti-inflammatory characteristics of bile acids (Duboc et al., 2013). Indeed, bile acids are known as anti-inflammatory mediators that inhibit NFκB activation and consequently reduce cytokine production by macrophages (Wang et al., 2011). This anti-inflammatory effect is characteristic of secondary bile acids but not the conjugated forms, which stresses the importance of deconjugation by gut microbiota (Duboc et al., 2013). Another anti-inflammatory effect of bile acids may be attributed to the activation of FXR, the bile acid receptors, which has been revealed as a protective factor against chemically induced colitis in mice (Gadaleta et al., 2011). Mice lacking FXR receptors have developed compromised intestinal barrier and antimicrobial defense in small intestine (Inagaki et al., 2006). The role of bile acids in protection against inflammation has been confirmed recently after identifying the association between a genetic variation of NR1H4, the gene encoding FXR receptor, and IBD (Attinkara et al., 2012). A pilot study of fecal bile acid and microbiota profiles in inflammatory bowel disease demonstrated that bile acid profiles were in general alike among patients with IBD and healthy controls (Vaughn et al., 2019). Oral administration of secondary bile acids in mice was reported to reduce the severity of colitis and ameliorate colitis-associated fecal dysbiosis at the phylum level (Van den Bossche et al., 2017). In accordance with the last study, dysbiosis of gut microbiota was found to induce deficiency in secondary bile acids in inflammatory-prone UC patients, which in turn leads to pro-inflammatory status in the intestine that may be treated via restoring secondary bile acids (Sinha et al., 2020). Like bile acid dysmetabolism, bile acid malabsorption was reported to be common reason of diarrhea in CD and colitis patients (Hou et al., 2018; Mena Bares et al., 2019).

### Oxidative Stress

Oxidative stress is thought to be one of the key players in the tissue damage associated with IBD. Oxidative stress is defined as an imbalance between reactive oxygen species and intracellular antioxidants. Hence, oxidative stress arises from either a higher production of oxidative free radicals (Keshavarzian et al., 2003) and/or deficient antioxidant machinery (Koutroubakis et al., 2004). The inflammatory cascade starts by the infiltration of proinflammatory cells to the intestinal mucosa, which release reactive oxygen species (ROS) and/or reactive nitrogen metabolites. For example, chemiluminescence analysis of ROS has elucidated a higher release of these free radicals by monocytes and polymorphonuclear cells extracted from both CD and UC biopsies (Kitahora et al., 1988; Keshavarzian et al., 1992). These free radicals induce more infiltration of proinflammatory cells and so this cycle is sustained and eventually causes tissue damage. The disruption of the intestinal epithelium exposes the immune system to the gut microbiota or other antigenic luminal components, which exacerbate the inflammation resulting in the active phenotype of the disease (Keshavarzian et al., 2003). For the cells to protect themselves against oxidative stress, they have to induce the production of antioxidant metabolites, which is not the case in IBD. It has been reported that IBD is associated with a depleted total antioxidant capacity of the cells or individual antioxidants. For example, IBD patients are characterized by a depletion of copper/zinc containing protein (superoxide dismutase and metallothionein), lower glutathione transferase activity with higher glutathione peroxidase in UC, and lower colonic ascorbate (Koutroubakis et al., 2004). Indeed, it is well reported that increased oxidative stress level in IBD patients and the detection of oxidative stress index rate could be used as predictors for the pathogenesis of IBD (Yuksel et al., 2017; Luceri et al., 2019). Bourgonje et al. (2019) showed that plasma free thiols are reduced in patients with CD, reflection of systemic oxidative stress, in clinical remission. The authors recommended that systemic oxidative stress and plasma free thiols may be a relevant therapeutic target and biomarker to monitor disease activity in CD (Bourgonje et al., 2019). Oral administration of probiotics in IBD patients were reported to effectiveness via the decrease of oxidative stress values (Ballini et al., 2019).

For the gut microbiota to maintain their homeostasis at this oxidative stress, they must develop oxidative stress resistance machinery. Morgan et al. (2012) demonstrated a shift of IBD microbiota toward microbes that possess the glutathione generation and reduction capability, which enables them to compensate for the oxidative stress. This machinery includes higher cysteine biosynthesis, which is a precursor of glutathione, riboflavin and NADPH, which are cofactors of glutathione reduction reaction and glutathione transfer gene (Morgan et al., 2012). Glutathione is a tripeptide (*γ*-glutamylcysteinylglycine) thiol that is produced by the majority of living cells (Anderson, 1998). The reduced form of glutathione protects the cells from toxic oxygen metabolites and other electrophiles via keeping the cell in a reduced state (Anderson, 1998). It also has other protective functions via regulation of gene expression, cell apoptosis and transport of organic solutes (Hammond et al., 2001). With regards to gut microbiota, glutathione has been reported to be biosynthesized by Proteobacteria members and a limited number of gram positive bacteria such as some Streptococcus spp. and *Staphylococcus aureus*, but not by *Clostridium*, *Bacillus* or *Micrococcus* members (Fahey et al., 1978). Also, glutathione sulfur transferase encoding genes that possess peroxidase activity are expressed by Proteobacteria members (Bartels et al., 1999). It is well documented now that IBD microbiota is dominated by Proteobacteria, which is known to produce some proinflammatory metabolites such as enterotoxins or LPS. This might generate a testable hypothesis that the inflammation associated stresses in the gut, such as oxidative stress, might constitute a selective pressure that induces a microbial shift toward the stress-resistant microbes. Furthermore, this shift might develop a colitogenic microbiota that could maintain the active chronic phenotype of the inflammation.

## Effect of IBD Medications on Gut Microbiota

Medications for IBD can be classified into five classes: aminosalicylates (sulfasalazine, olsalazine, and mesalamine), Corticosteroids (cortisone, prednisone, prednisolone, hydrocortisone, methylprednisolone, beclometasone, and budesonide), immunosuppressive agents (6-Mercaptopurine, azathioprine, methotrexate, and tacrolimus), antibody agents (Anti-TNF agents (infliximab, adalimumab, certolizumab pegol), and antibiotics (metronidazole, ciprofloxacin and rifaximin) (Gade et al., 2020; Targownik et al., 2020). The choice of the treatment strategy depends on the severity of the disease and the response to previous therapy. The effect of some of the above-mentioned drugs on gut microbiota are summarized in Table 2. Interestingly some drugs that are used for treatment of IBD requires metabolic activation via the gut microbiota e.g., sulfasalazine, balsalazide (mesalamine prodrug), olsalazine, and methotrexates (Crouwel et al., 2020).

**TABLE 2:** The effect of medications used for treatment of IBD on gut microbiota.

**Drug**	**Reported effects on gut microbiota diversity and composition**	**Model/Disease subtype**
Sulfasalazine	• Restores gut diversity	Rats/Colitis
	• Increases SCFAs- producers and lactic acid-producers	
	• Decreases Proteobacteria (Zheng H. et al., 2017).	
5- Aminosalicylates	• Positively alters the diversity, composition, and bacterial interaction patterns.	Human mucosa/UC
	• Decreases *Escherichia*–*Shigella* (Xu et al., 2018).	
Mesalamine	• Partially restores gut microbiota diversity and composition	Human/UC
	• Significant alteration of 129 correlated metabolites (Dai et al., 2020).	
	• Increase the abundance of *Erysipelotrichaceae* species (Vich Vila et al., 2020).	
Oral steroids	• Increases the abundance of *Methanobrevibacter smithii* (Vich Vila et al., 2020).	Human/IBD
Azathioprine or mercaptopurine	• Inhibits the growth of *Campylobacter concisus* and other enteric microbes that are associated with IBD (Liu et al., 2017).	
Azathioprine or anti-TNF antibodies	• Restoration of intestinal microbial	Human/CD
	• Decrease in Proteobacteria	
	• Increase of Bacteroidetes (Effenberger et al., 2021).	
Metronidazole	• Increases the abundance of Bifidobacteria (Particularly Bifidobacterium pseudolongum) and enterobacteria (Pelissier et al., 2010).	Rats/Colitis
	• Allowed the retention of a beneficial microbiota that reduced the severity of colitis upon fecal microbiota transplantation in an experimental colitis model (Strati et al., 2021).	
Ciprofloxacin	• Increased relative abundance of *Bacteroides* and Firmicutes genera *Blautia*, *Eubacterium* and *Roseburia* (Stewardson et al., 2015).	Human/urinary tract infection.
	• Reduced *Bifidobacterium*, *Alistipes* and 4 genera from the phylum Firmicutes (*Faecalibacterium*, *Oscillospira*, *Ruminococcus* and *Dialister*) (Stewardson et al., 2015).	
Rifaximin	• Inducing the growth of bacteria useful to the host without changing its general composition (Ponziani et al., 2017).	

It should be mentioned that some IBD medications are reported to affect either the metabolism of gut microbiota or the metabolic status of intestinal cells by altering the intestinal biota. Sulfasalazine was reported to enhance carbohydrate metabolism, citrate cycle and decrease the oxidative stress (riboflavin, sulfur, cysteine) (Zheng H. et al., 2017). Dahl et al. (2017) showed that, mesalamine was able to decrease polyphosphate levels in bacteria, including members of the human gut microbiota. This reduction leads to bacterial sensitization to oxidative stress and decreases bacterial colonization (Arthur et al., 2012). Effenberger et al. (2021) performed an *in-silico* metabolic prediction analysis by including azathioprine or anti-TNF antibodies-treated IBD groups and assessed the effect of gut microbiota function on remission status. They found that the predicted butyrate synthesis was significantly enriched in patients achieving clinical remission. The use of oral steroids in IBD patients was demonstrated to affect two biosynthetic pathways of methanogenesis and one pathway in the biosynthesis of vitamin B2 and nucleosides (Vich Vila et al., 2020). The oral administration of metronidazole was found to reduce basal oxidative stress in colonic tissue of healthy rat (Pelissier et al., 2007) and increase the thickness of colonic mucosal layer by about twofolds (Pelissier et al., 2010). Moreover, the metronidazole-treated microbiota in murine fecal donors retained its ability to control inflammation co-occurring with enrichment of *Lactobacillus* and innate immune responses including invariant natural killer T cells in experimental colitis (Pelissier et al., 2010).

## Therapeutic Strategies of IBD Targeting Gut Microbiome

### Probiotics, Prebiotics, and Postbiotics

Probiotics are living organisms that when are given in appropriate amount to the host result in health benefit (Hill et al., 2014). Prebiotics are substances, which are utilized by probiotics leading to enhancing the health (Gibson et al., 2017). Postbiotics are non-living microorganisms with or without their cell components and metabolites that confer health benefits (Salminen et al., 2021). Although probiotics have the ability to modulate microbiome composition resulting in enhancing the growth of good species and inhibiting the growth of pathogenic ones, their use in IBD treatment is recommended only in the context of clinical trials in adults and children (Su et al., 2020). Probiotics have anti-inflammatory impact and enhance intestinal barrier functions (Abraham and Quigley, 2017). In adults and children with pouchitis, the use of eight-strain combination of *Lactobacillus plantarum*, *Lactobacillus paracasei* subsp*paracasei*, *Lactobacillus delbrueckii* subsp *bulgaricus*, *Lactobacillus acidophilus*, *Bifidobacterium breve*, *Bifidobacterium longum* subsp. *longum*, *Bifidobacterium longum* subsp. *infantis*, and *Streptococcus salivarius* subsp. *thermophilus* is recommended (Su et al., 2020). It is well documented that the probiotic cocktail VSL#3, which is composed of 3 Bifidobacteria strains, 4 lactobacilli strains, and 1 *Streptococcus* strain, is promising for treatment of patients suffering from IBD (Bibiloni et al., 2005; Miele et al., 2009; Fedorak et al., 2015). Also, the probiotic *Lactobacillus reuteri* ATCC 55730 was speculated to be helpful during the treatment of IBD cases (Oliva et al., 2012). In addition, the probiotic cocktail composed of *Lactobacillus acidophilus* NCIMB 30175, *Lactobacillus plantarum* NCIMB 30173, *Lactobacillus rhamnosus* NCIMB 30174, and *Enterococcus faecium* NCIMB 30176 has a well-documented effect in the treatment of IBD patients especially UC (Bjarnason et al., 2019; Moens et al., 2019). More recently, next generation probiotics such as *A. muciniphila* and *F. prausnitzii* and their supernatants (postbiotics) are reported to exhibit beneficial effects during IBD treatment (Sokol et al., 2008b; O’Toole et al., 2017). Fermented foods are good sources of probiotics including miso, tempeh, kefir, kimchi, pickled vegetables, yogurt with live active cultures, kombucha tea and sauerkraut (Sultan et al., 2020).

Also, prebiotics such as inulin, resistant starch, gums, pectins, and fructo-oligosaccharides are reported to be beneficial in the treatment of IBD patients through enhancing functions of the intestinal barrier and protecting vs. invasion and translocation of pathogens (Akram et al., 2019). Although there are many commercial products of these prebiotic fibers, healthy diets are considered their main source such as bananas, beans, onions, raw version of leeks, oats, dandelion greens, wheat, garlic, asparagus, artichokes, barley, seaweed, and other fruits and vegetables that are rich in fibers and indigestible carbohydrates (Sultan et al., 2020). Such prebiotics aid the growth of normal gut microbiota and the production of SCFAs, which results in enhancing the activity of immune cells, maintaining the levels of glucose and cholesterol as well as decreasing the pH of the colon, which results in enhancing the condition (Akram et al., 2019; Sultan et al., 2020).

### Phage Therapy

Phages are the most ubiquitous organisms worldwide and they are characterized by their selectivity and specificity to bind their target host (Abd El-Aziz et al., 2019). Phages can bind and lyse specific bacterial strains within certain species (El-Mowafy et al., 2021). This capability gives the phage the advantage to be safer during the treatment of bacterial infections than commonly used antibiotics and to have limited effect on microbiota of the host (Abd El-Aziz et al., 2019; El-Mowafy et al., 2021).

The use of Russian coliphage or oral T4-like coliphages in children with bacterial infection-induced diarrhea does not induce any side effect, however, it was unsuccessful to enhance the conditions (Sarker et al., 2016). Recently, it is recommended to use phages against AIEC in patients suffering from IBD (Galtier et al., 2017). AIEC is an abnormal pathogen, which is commonly found in the ileal mucosa of IBD patients (Barnich et al., 2007). The use of bacteriophages to lyse AIEC significantly reduced the symptoms of DSS-induced colitis in transgenic mice expressing the human receptor for AIEC named CEACAM6 (Galtier et al., 2017). Therefore, phages targeting AIEC may be a promising therapeutic approach for the treatment of IBD patients.

### Fecal Microbiota Transplantation

FMT is defined as the transplantation of healthy donor fecal microbiota into the gut of a patient as a trial to reverse the dysbiosis, restore homeostasis of the gut microbiota and improve the condition (Burrello et al., 2018; Hvas et al., 2019). This approach was reported to be successful during the treatment of recurrent *C. difficile* infection (CDI) (Khoruts and Sadowsky, 2016). The successful treatment of IBD patients using FMT depends on three main factors including; early treatment, content of fecal matter of the donor and usage of multiple FMTs (Moayyedi et al., 2015). Treatment of IBD patients using FMT is based on the idea that regaining the gut microbiota homeostasis will alter the growth of pathogens like *C. difficile* that are responsible for the disease (Khoruts et al., 2016). This is achieved through competition between the regained microbiota and pathogens for nutrients and colonization, production of antimicrobials by regained microbiota that directly affect pathogens, and vegetative growth and spore germination inhibition through bile acid mediated mechanism (Khoruts and Sadowsky, 2016). However, it is noteworthy that the fungal and viral content of the stool of donors may affect the outcome of IBD treatment using FMT (Draper et al., 2018; Zuo et al., 2018). It is reported that using stool rich in *C. albicans* significantly decreases the treatment efficacy (Zuo et al., 2018). Moreover, it was depicted that following FMT, different patients who received fecal matter from the same donor exhibited extremely individualized virus colonization patterns (Draper et al., 2018).

Costello et al. (2019) showed that 1 week treatment with anaerobically prepared fecal material, in mild to moderate UC patients, led to a higher probability of remission after 8 weeks. Another interesting work followed a randomized controlled study to investigate the role of FMT to maintain remission in CD patients (Sokol et al., 2020). The authors found a low similarity index between donor and recipient microbiota in some patients suggesting that a single FMT might not be enough to stimulate significant changes in these patients. An interesting study aimed to investigate the optimum timing of FMT for maintaining the long-term clinical benefits in UC (Li et al., 2020). The authors demonstrated that UC patients could take the 2nd course of FMT within 4 months after the initial course of FMT, which will allow clinicians to consider sequential FMTs as a long-term treatment strategy for UC. Moreover, they showed that the relative abundance of *Eubacterium*, *Ruminococcus*, *Eggerthella*, and *Lactobacillus* in UC patients can be used as predictors for the long-term efficacy of FMT (Li et al., 2020). Similarly, the species *Eubacterium hallii, Ruminococcus bromii, and Roseburia inulivorans* were recommended to predict the success of FMT therapy in UC patients (Paramsothy et al., 2019). However, the application of FMT has many disadvantages. First, different samples represent different bacterial ensembles which means variable efficacy and the outcome will rely on the sample (Choi and Cho, 2016). Additionally, safety risks may arise from the presence of undesirable strain/functionality within the donated stool sample such as transferable antibiotic resistance elements, virulence factors or pathogenic strains enteropathogenic *E. coli* (Nataro and Kaper, 1998; Choi and Cho, 2016).

### Dietary Interventions to Modulate the Gut Microbiota in Inflammatory Bowel Diseases Patients

There is a tight bond between diet, gut microbiota, colonocytes, and immune cells during both the intestinal healthy conditions (homeostasis) and inflammation (Celiberto et al., 2018). Healthy diet patterns including diets rich in fruits, vegetables, probiotics, fermentable food, fibers, prebiotics, and adequate concentrations of vitamin D are good for intestinal health and homeostasis (Rinninella et al., 2019). Healthy diets generally result in more microbial diversity and an increase of good bacteria including *F. prausnitzii*, *Bifidobacterium* spp., and *Lactobacillus* spp. that result in increased production of SCFAs, especially butyrate (Celiberto et al., 2018; Rinninella et al., 2019). Butyrate has strong anti-inflammatory characters as it improves the function of intestinal barrier and endorses the proliferation of regulatory T cells (Treg) (Kespohl et al., 2017). Moreover, butyrate promotes the dendritic cells (DC) within the lamina propria and commensal microbiota antigens within the intestinal lumen. Dendritic cells mainly trigger Treg cells to produce transforming growth factor-β (TGF-β) and interleukin-10 (IL-10) through the production of TGF-β resulting in tolerant immune response and intestinal homeostasis (Zheng L. et al., 2017). These modulations result in more thick mucus layer, which is an important property of intestinal homeostasis, giving a protective shield between epithelial cells and luminal bacteria (Butzner et al., 1996; Segain et al., 2000; Geirnaert et al., 2017). Moreover, healthy diets represent a good source of aryl hydrocarbon receptor (AhR) which induce the production of IL-22, via the innate lymphoid cell 3 (ILC3) that maintains intestinal barrier function (Song et al., 2020).

Healthy diets harboring adequate concentrations of vitamin D as well as vitamin D supplements are reported to be helpful during IBD cases (Celiberto et al., 2018). It is reported that vitamin D is crucial for maintaining the composition of gut normal microbiota, where vitamin D receptor (VDR) deficient mice exhibited increased abundance of Bacteroidetes and Proteobacteria as well as diminished *Lactobacillus* spp. resembling the dysbiotic cases of IBD patients (Winter et al., 2017; Windsor and Kaplan, 2019). Vitamin D enhances the epithelial barrier function and promotes the production of antimicrobial peptides (Celiberto et al., 2018). It also promotes the proliferation of dendritic cells, which in turn promotes the production of the immunosuppressive IL-10 through provoking Treg cells (Sokol et al., 2006b; Jin et al., 2015). Vitamin D also decreases the production of pro-inflammatory cytokines including TNF-γ, IL-17, and IL-21 through its inhibitory effect on IL-12 and IL-23 that are responsible for the responses of Th-1 and Th-17, respectively (Santos-Antunes et al., 2016; Winter et al., 2017).

On the other hand, unhealthy diets such as Western dietary patterns those are low in fruits, vegetables, probiotics, fermentable food, fibers, prebiotics, and vitamin D are directly related to dysbiosis of the microbiota and diminished microbial diversity, decreasing SCFAs production, reducing good bacteria and increasing the pathobionts such as *E. coli* and *Clostridium difficile* (Sugihara and Kamada, 2021). This results in more patchy and thinner mucus layer, which as a result provides less protection between colonocytes and luminal bacteria (Sugihara and Kamada, 2021). In addition, the compromised intestinal barrier function, due to decreased expression of cellular tight junctions, leads to escape of harmful bacterial products including lipopolysaccharides (LPS) from the intestinal lumen to the lamina propria (Ghosh et al., 2020). LPS trigger the macrophages via binding to toll-like receptors (TLR) leading to the production of TNF-α. TNF-α endorses the proliferation of T helper cell type 1 (Th1) that produce the proinflammatory cytokines including TNF-α and TNF-γ resulting in inflammation and dysfunctionality of the intestinal barrier (Grassin-Delyle et al., 2020). Moreover, the condition may worsen due to decreasing Treg cells responsible for the production of IL-10, which mitigates the intestinal inflammation (Iyer and Cheng, 2012).

## Conclusion

**FIGURE 2:**
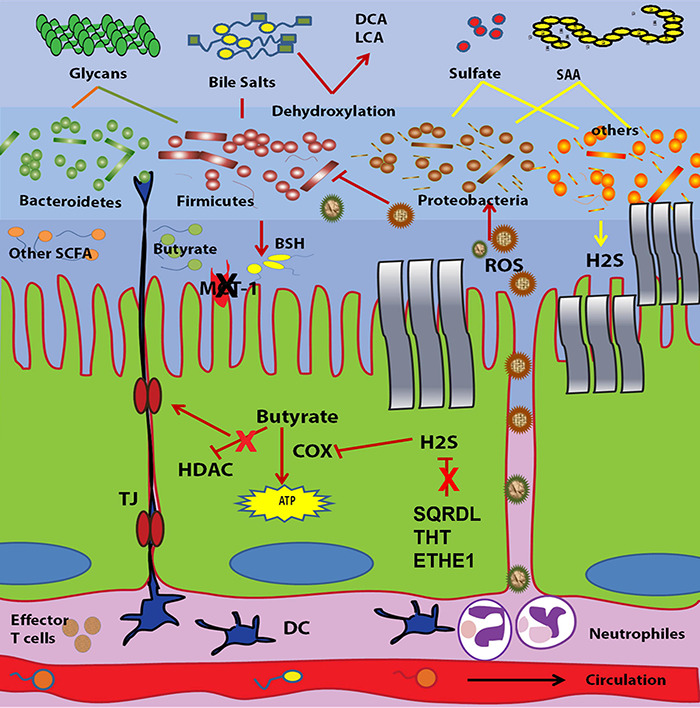
Proposed Host-microbe metabolic dysfunctionalities in inflammatory bowel disease. The oxidative stress environment created by inflammation acts as a selective pressure. This pressure favors the microbes that are able to resist that stress such as Proteobacteria members but not Firmicutes members. This in turn leads to less short chain fatty acids (SCFAs) production, less bile salts hydrolases (BSHs) in association with higher H_2_S release. From the host side, impaired butyrate transport and oxidation, less mucin secretion, low tight junction (TJ) expression, and defective H_2_S detoxification are the major host metabolic perturbations associated with IBD.

From what has been discussed above, gut microbiota exerts many beneficial roles to human health, where any permanent disturbance of this ensemble could metabolically lead to a disease state. Gut microbiota of IBD patients is characterized by depletion of Firmicutes and Bacteroidetes with increased abundance of Proteobacteria and Actinobacteria. These changes in microbial composition result in perturbations of microbial functions that could be summarized in less short chain fatty acid production, less bile acid hydrolysis, higher redox potential and increased H_2_S production (Figure 2). This pattern of microbial metabolic perturbations is associated with defectiveness of some human cellular pathways such as defective H_2_S detoxification, SCFAs transport and oxidation, and high luminal redox potential. The interaction between both sides of dysfunctionality is considered pathogenic to the host and might modulate the chronicity of the disease. However, the knowledge of the cause/effect relationship between these metabolic perturbations and the inflammation has so far been limited. In addition, the actual molecular mechanisms that regulate the interaction between both sides of the equation still need to be identified. Still, different strategies are being developed to manipulate gut microbiota to reduce the intestinal inflammation and improve the disease outcomes. These include live biotherapeutics such as FMT and probiotics. Taking in account the disadvantages of FMT and enrichment-based approaches in general, new approaches may rely on bottom-up rational to design identified bacterial consortia that are metabolically interdependent and exert a variety of protective functions to the host and the gut microbiota. These functions include generation of beneficial metabolites such as SCFAs and indole, deconjugation and conversion of bile acids, competition for critical nutrients and synthesis of antimicrobial molecules against opportunistic pathogens.

## Author Contributions

All authors listed have made a substantial, direct and intellectual contribution to the work, and approved it for publication.

## Conflict of Interest

The authors declare that the research was conducted in the absence of any commercial or financial relationships that could be construed as a potential conflict of interest.

## Publisher’s Note

All claims expressed in this article are solely those of the authors and do not necessarily represent those of their affiliated organizations, or those of the publisher, the editors and the reviewers. Any product that may be evaluated in this article, or claim that may be made by its manufacturer, is not guaranteed or endorsed by the publisher.
